# Possible role of *Toxoplasma gondii* in brain cancer through modulation of host microRNAs

**DOI:** 10.1186/1750-9378-8-8

**Published:** 2013-02-08

**Authors:** Sivasakthivel Thirugnanam, Namita Rout, Munirathinam Gnanasekar

**Affiliations:** 1 Department of Biomedical Sciences, University of Illinois, College of Medicine, 1601 Parkview Ave, Rockford, IL, 61107, USA; 2 New England Primate Research Center, Harvard Medical School, Southborough, MA, 01772, USA

**Keywords:** *T. gondii*, Brain cancer, miRNA

## Abstract

**Background:**

The obligate intracellular protozoan parasite *Toxoplasma gondii* infects humans and other warm-blooded animals and establishes a chronic infection in the central nervous system after invasion. Studies showing a positive correlation between anti-*Toxoplasma* antibodies and incidences of brain cancer have led to the notion that *Toxoplasma* infections increase the risk of brain cancer. However, molecular events involved in *Toxoplasma* induced brain cancers are not well understood.

**Presentation of the hypothesis:**

*Toxoplasma* gains control of host cell functions including proliferation and apoptosis by channelizing parasite proteins into the cell cytoplasm and some of the proteins are targeted to the host nucleus. Recent studies have shown that *Toxoplasma* is capable of manipulating host micro RNAs (miRNAs), which play a central role in post-transcriptional regulation of gene expression. Therefore, we hypothesize that *Toxoplasma* promotes brain carcinogenesis by altering the host miRNAome using parasitic proteins and/or miRNAs.

**Testing the hypothesis:**

The miRNA expression profiles of brain cancer specimens obtained from patients infected with *Toxoplasma* could be analyzed and compared with that of normal tissues as well as brain cancer tissues from *Toxoplasma* uninfected individuals to identify dysregulated miRNAs in *Toxoplasma-*driven brain cancer cells. Identified miRNAs will be further confirmed by studying cancer related miRNA profiles of the different types of brain cells before and after Toxoplasma infection using cell lines and experimental animals.

**Expected outcome:**

The miRNAs specifically associated with brain cancers that are caused by *Toxoplasma* infection will be identified.

**Implications of the hypothesis:**

*Toxoplasma* infection may promote initiation and progression of cancer by modifying the miRNAome in brain cells. If this hypothesis is true, the outcome of this research would lead to the development of novel biomarkers and therapeutic tools against *Toxoplasma* driven brain cancers.

## Background

Chronic *Toxoplasma gondii* infection is one of the most prevalent parasitic infections in humans worldwide and nearly one-third of the population has been estimated to be carrying the parasite [1,2]. Upon entry, *T. gondii* transforms into fast replicating tachyzoites and infects various organs of the body including the central nervous system (CNS). To evade host immune response, some of the tachyzoites differentiate in to bradyzoites, which are slow growing and form tissue cysts in the brain [3,4]. During chronic infection, *T.gondii* tissue-cysts persist for lifetime of the host without provoking any host immune attack [5].

Host cell invasion is an active process which is essential for survival and replication of parasites. While invading a host cell, *T. gondii* discharges proteins from its secretory organelles which include micronemes, rhoptries, and dense granules. Detection of parasitic proteins with kinase and phosphatase domains in the host nucleus suggests that the parasite modulates the host cell signaling and gene expression [6]. This notion is further supported by a recent finding that *Toxoplasma* infection orchestrates the expression of host miRNAs which are deliberated as the key regulators of signaling pathways [7].

MicroRNAs (miRNAs) are short (19–24 nucleotides) non-protein coding RNAs endogenously regulate gene expression at the post-transcriptional level by binding with target mRNAs that trigger their degradation and/or translational inhibition. A single miRNA can regulate multiple mRNAs; therefore, miRNAs have imperative effects on cell signaling networks [8,9]. Several studies have identified differential expression of miRNAs in brain tumors including glioblastoma, pituitary adenoma, and medulloblastoma when compared to normal tissues [10,11]. The miRNAs play a critical role in brain carcinogenesis and metastasis by acting as either oncogenes or tumor suppressors [12].

*Toxoplasma* is an important non-viral pathogen shown to be associated with the occurrence of brain tumors. Previous investigations have revealed that *T. gondii* could cause gliomas in experimental animals [13]. Studies carried out by Ryan et. al., [14] showed that antibody positivity to *Toxoplasma* is associated with meningioma. An epidemiological study analyzing data from 37 countries for the incidences of adult brain cancers and *Toxoplasma* infected people associated a nearly two-fold increase in the risk of brain cancers across the range of prevalence in Toxoplasma infection [15]. These studies, though correlational, suggest that *Toxoplasma* should be investigated further as a possible oncogenic pathogen in humans. A recent work conducted in France showed that mortality rates due to brain cancer correlated positively with the local sero-prevalence of *Toxoplasma*, particularly in the people who are 55 years of age or older [16]. Despite these strong evidences suggesting that *Toxoplasma* is associated with brain cancer, it is unclear how the infection causes this debilitating cancer in humans. In this article, we present a hypothesis that *Toxoplasma* infection may have the ability to modulate the host miRNAs and could potentially promote the development of brain cancer.

### Presentation of the hypothesis

*Toxoplasma* has an inherent ability to manipulate host cell signaling pathways and processes by interfering with the global gene expression profiles of the invaded cells [6,17]. Microarray analysis showed that more than 1,000 host cell genes involved in the various processes including apoptosis, inflammation, metabolism, cell growth and differentiation, are up-regulated or down-regulated after the *Toxoplasma* invasion [18-20]. During intracellular infections, the host cell responds by initiating apoptotic response which reduces survival and proliferation of the parasites and makes the parasites susceptible to immune attack. However, *Toxoplasma* has established several strategies to neutralize the extrinsic and intrinsic cellular suicide programs of the infected cells [6,21]. Invasion of *Toxoplasma* turns host cells resistant to multiple inducers of apoptosis, including Fas-dependent and Fas-independent CTL-mediated cytotoxicity, IL-2 deprivation, gamma irradiation, UV irradiation, and calcium ionophorebeauvericin [22]. *Toxoplasma* exerts different anti-apoptotic mechanisms for the successful establishment in different cell types [6,21,22]. *Toxoplasma* significantly reduces Fas/CD95-triggered apoptosis by impairing activation of the initiator caspase 8 in type I cells [23]. While in type II cells, *Toxoplasma* targets activation of the pro-apoptotic Bax and Bak to inhibit the apoptogenic function of mitochondria [24]. *Toxoplasma* infection has been shown to promote the expression of anti-apoptotic proteins: Bcl2, Bfl1, Bcl-Xl, Bcl-w, Mcl-1, Bad and Bax in host cells [25-27]. *Toxoplasma* also modulates several cell signaling pathways including AKT and Phosphoinositide 3-kinases (PI3Ks) pathways [28,29].

In a search to identify the parasite effector molecules, proteins of rhoptry and dense granule secretory organelles were found to be secreted by the parasite during the invasion, that are capable of modulating host signaling pathways [6]. Interestingly, recent studies showed that miRNAs, which are important regulators of gene expression, are manipulated by *Toxoplasma* to interfere with the host cell functioning [7,30]*.* In addition, *Toxoplasma* infection has been shown to specifically increase levels of mature miR-17-92 derived miRNAs in primary human foreskin fibroblasts [7]. *Toxoplasma* dependent up-regulation of the miR-17-92 promoter is at least partly responsible for this increase.

The miR-17-92 cluster is associated with brain cancers [31,32]. Primary human astrocyticglioma tissue specimens are found to be over-expressing the miR-17-92 cluster, compared to non-neoplastic brain control tissues [31]. Inhibition of the miR-17-92 results in reduced cell viability and cell proliferation and increased apoptotic rates [33]. Furthermore, expression analysis of 90 primary human medulloblastomas revealed that components of the miR-17-92 polycistron are greatly up-regulated miRNAs in the most common malignant pediatric brain tumor, which is medulloblastoma, and miR-17-92 expression correlates with high levels of MYC family proto-oncogenes. Besides, expression analysis of the miR-17-92 cluster showed that three miRNAs (miR-92, miR-19a, and miR-20) are over-expressed only in human medulloblastomas with a constitutively activated Sonic Hedgehog/patched (SHH/PTCH) signaling pathway, suggesting the role of the miR-17-92 cluster in the formation of aberrant SHH/PTCH pathway driven medulloblastomas [32]. Molecular targets of miR-17-92 are found to be CDKN1A, BCL2L11, PTEN and E2F1; and depletion of miR-17-92 results in the derepression of CDKN1A and E2F1 at the mRNA level and of E2F1 and PTEN at the protein level [31]. Since the AKT pathway has been shown to be activated by *Toxoplasma* infection [6], an interesting possibility is that miR-17-92 mediated decrease in levels of PTEN during *Toxoplasma* infection in brain cells could be activating AKT pathways which may result in the development of brain cancer.

In addition to miR-17-92, various other miRNAs are critically involved in development and progression of different types of brain cancers [11,34]. Glioblastomamultiforme (GBM), the most common and aggressive primary intracranial malignancy of brain tumors, is shown to be associated with up-regulation of miR-221 and down-regulation of miR-128, miR-181a, miR-181b, and miR-181c [35]. Another miRNA elevated in GBM is miR-21, which regulates multiple genes associated with cancer cell proliferation, such as, apoptosis, and invasiveness [36]. Also, miR-21 is up-regulated in medulloblastoma cells and its down-regulation increased the expression of negative modulators of cancer cell migration, E-Cadherin and TIMP2 proteins and their positive regulator PDCD4 which results in decreased motility of medulloblastoma [37,38]. Pituitary adenomas are the most common tumors of the central nervous system and show differential expression of 30 miRNAs including tumor suppressors belonging to the let-7 family, compared to normal pituitary gland [39].

**Figure 1:**
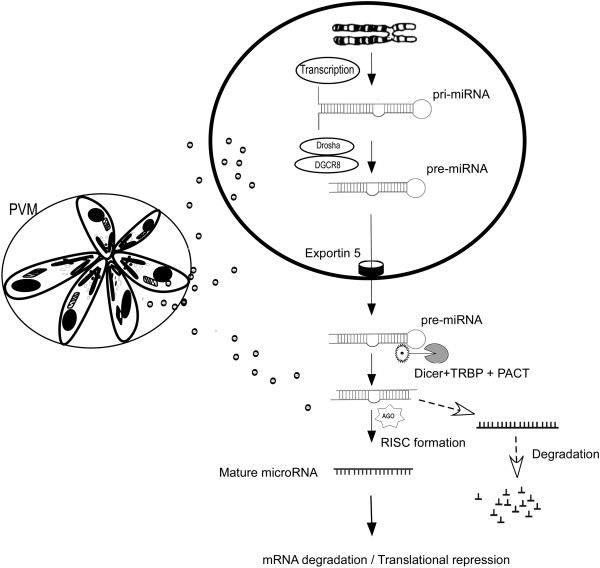
**Schematic illustration of the hypothesis that *Toxoplasma *infection alters miRNA pathway leading to brain carcinogenesis. **The miRNA synthesis pathway is mediated by multiple protein complexes that sequentially cleave, export and incorporate miRNA into the silencing machinery. miRNA is transcribed by RNA polymerase II or III and this pri-miRNA is processed by Drosha–DGCR8 (Pasha) complex to pre-miRNA, which is exported from the nucleus by Exportin-5 to the cytoplasm. The RNase Dicer is associated with the double-stranded RNA-binding protein TRBP and the PKR activator PACT processes the pre-miRNA hairpin to 22-nt miRNA duplexes. One strand of mature miRNA is loaded into the RNA-induced silencing complex (RISC) along with Argonaute (Ago2) proteins and it directs RISC to silence target mRNAs through mRNA cleavage or translational repression while the complementary strand is degraded. This pathway is tightly regulated at transcriptional and post transcriptional level. The miRNA stability and post translational modifications are used to modulate the functionality of the miRNAs. Proteins of miRNA processing complexes also play a crucial role in regulating the miRNA processing pathway. We hypothesize that effector molecules released by *Toxoplasma* into the host cell may interfere with miRNA synthesis and maturation pathway, which in turn modulate host cell survival or death signaling pathways.

Furthermore, the *Toxoplasma* genome codes for ostentatious RNA silencing machinery and endogenous small silencing RNAs, including miRNAs [40]. *Toxoplasma* may be using its own miRNAs to modify host cell functions, analogous to some mammalian viruses encoding their own miRNAs [41]. In view of above findings, we hypothesize that *Toxoplasma* associated miRNA dysregulation may be playing a central role in the development of brain carcinogenesis, considering the significance of miRNAs in brain tumor development and progression [Figure 1.

To our knowledge, there is only one study that utilized primary human foreskin fibroblasts to elucidate the effect of *Toxoplasma* infection on the host cell miRNAs [7]. However, the host miRNAs regulated by *Toxplasma* may vary based on cell type. Thus the possibility that *Toxoplamsa* infection can alter expression of several other miRNAs along with miR-17-92 in different types of host cells cannot be ruled out. Therefore, it is essential to study miRNA profiles of various types of Toxoplasma positive brain cancer cells; especially GBM or medulloblastoma, the most prevalent brain cancers.

### Testing the hypothesis

**Figure 2:**
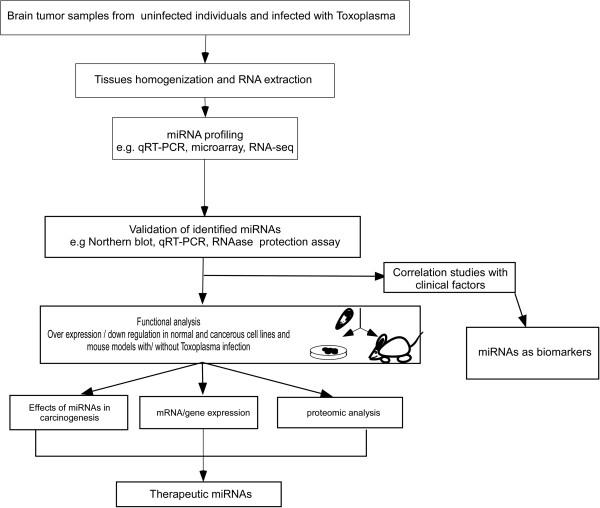
**Experimental approach validating the hypothesis. **Flowchart of suggested procedures required to identify and validate miRNAs associated with *Toxoplasma-*mediated brain cancers. Aberrantly expressed miRNAs will be identified by analyzing miRNA expression profiles of specimens obtained from *Toxoplasma* infected brain cancer patients in comparison to the uninfected brain cancer specimens and specimens from normal individuals. The identified miRNAs will be further validated in brain cell lines and animal models by experimental *Toxoplasma* infection. Functional analyses will be helpful to identify miRNAs and their potential targets that regulate host signaling pathways. Thus, identification and validation of miRNAs will lead to the development of biomarkers and therapeutics for *Toxoplasma* associated brain cancers.

To test this hypothesis, miRNA profiles of normal brain and cancer tissues collected from brain cancer patients with and without *Toxoplasma* infection will be first studied. The details of the study have been summarized in Figure 2. Frozen, fresh or formalin-fixed paraffin-embedded brain tissues will be obtained from brain banks and hospitals performing brain surgeries and examined by pathologists, assessing the presence of *Toxoplasma* infection and the proportion of normal and tumor cells. Commercially available reagents containing concentrated chaotropic salts, such as guanidiniumthiocyanate (e.g., Trizol) followed by silica column extraction will be used to extract high quality miRNA from brain tissue samples. Three major approaches are now commonly used for miRNA profiling: quantitative reverse transcription PCR (qRT-PCR), microarrays and high-throughput RNA sequencing. Additional new methods are emerging which will be applied for miRNA profiling in the future [42]. High throughput microarray technology will be used to identify miRNA signatures associated with *Toxoplasma* infected brain cancer cells. The identified global miRNAs profile will be further validated by qRT-PCR in different brain cell lines and brain cells of experimental animals before and after *Toxoplasma* infection. In addition, brains of the animals chronically infected with Toxoplasma will be monitored *in vivo* for malignant transformation and tumor growth using neuroimaging techniques [43,44]. The miRNAs that are differentially expressed in *Toxoplasma* driven brain cancer cells could be used as markers to classify these cancers. As briefly outlined, in Figure 2, functional analysis of identified miRNAs by over expression and down regulation in cell lines and experimental animals will reveal the effect of *Toxoplasma-*modified miRNA expression on survival and death pathways of brain cancer cells. Thus, these studies could be very useful in the development of miRNA-based therapeutics.

### Implications of the hypothesis

Decades of *Toxoplasma* research has made us aware of the parasite’s ability to manipulate host cell signaling pathways. Though some parasite proteins were identified as effector molecules for this function, the underlying molecular mechanisms of *Toxoplasma-* mediated brain carcinogenesis are not clearly understood. Our hypothesis predicts that *Toxoplasma-*modified miRNAs may play a critical role in initiation and progression of brain carcinogenesis, though the outcome of the infection possibly depends on the mode of infection, parasitic strain, type of host cell and miRNA expression patterns of host cell and parasite proteins. If our hypothesis is true, miRNAs critically involved in the *Toxoplasma* driven cancers could be identified and they could be utilized as novel diagnostic and therapeutic targets. Thus, further research on the specific miRNA pathways affected by *Toxoplasma* in various brain cells would open new avenues in the diagnosis and treatment of brain cancers caused by *Toxoplasma* infection.

## Competing interest

The authors declare that they have no competing interests.

## Authors’ contributions

ST, NR and MG developed the hypotheses and contributed to writing the manuscript. All authors read and approved the final manuscript.
